# Efficient expression of enterovirus 71 based on virus-like particles vaccine

**DOI:** 10.1371/journal.pone.0210477

**Published:** 2019-03-07

**Authors:** Hye-Jin Kim, Ho Sun Son, Sang Won Lee, Youngsil Yoon, Ji-Yeon Hyeon, Gyung Tae Chung, June-Woo Lee, Jung Sik Yoo

**Affiliations:** 1 Division of Vaccine Research, Center for Infectious Diseases, Korea National Institutes of Health, Korea Centers for Disease Control and Prevention, Osong-eup, Cheongju, Chungcheongbuk-do, Republic of Korea; 2 Division of Strategic Planning for Emerging Infectious Disease, Centers for Disease Control and Prevention, Osong, CheongJu, Chungcheongbuk-do, South Korea; 3 Division of Viral Disease, Center for Laboratory control of Infectious Disease, Centers for Disease Control and Prevention, Osong, CheongJu, Chungcheongbuk-do, South Korea; Icahn School of Medicine at Mount Sinai, UNITED STATES

## Abstract

Enterovirus (EV) 71 is the main pathogen associated with hand-foot-mouth disease (HFMD) and can lead to the disease with severe mortality in children. Since 2009, in the Republic of Korea, an outbreak of EV71 C4a infection with neurologic involvement emerged, where in HFMD involvement was identified and central nervous system complications were reported. In this study, EV71 C4a virus-like particles (VLPs) produced by recombinant technology were generated in a baculovirus expression system. To improve the production yield, EV71 VLP was constructed using the dual promoter system baculovirus P1 and 3CD (baculo-P1-3CD), which harbored both the structural protein-encoding P1 region under the control of the polyhedron promoter and the 3CD protease gene under the regulation of the *CMV-IE*, *lef3*, *gp41*, or chitinase promoters to augment the level of gene transcription. Efficient VLP expression was demonstrated through optimization of incubation time and insect cell type. In addition, to evaluate the potential of VLP as a vaccine candidate, we tested the neutralizing antibodies and total anti-EV71 IgG from the purified EV71 C4a VLP serum. The recombinant EV71 VLP exhibited the morphology of self-assembled VLP, as determined by electron microscopy. Use of baculo-P1-3CD-gp41 led to a high yield (11.3mg/L < 40kDa) of VLPs in High-Five^TM^ cells at 3 days post-infection. Furthermore, the potential of VLP as a vaccine was evaluated through the neutralizing ability elicited by the purified EV71 VLP after immunization of BALB/c mice, which was shown to induce potent and long-lasting humoral immune responses as evidenced by the cross-neutralization titer. Our results could be used to expedite the developmental process for vaccines under clinical trials and to ensure manufacturing consistency for licensing requirements.

## Introduction

Enterovirus 71 (EV71) is a non-enveloped virus with a positive-stranded RNA genome consisting of three regions [1]. The P1 precursor region encodes the structural proteins, whereas the P2 and P3 regions encode nonstructural proteins including the 3CD protease that cleaves P1 into four viral shell proteins [2]. Based on VP1 sequence alignment, EV71 is currently classified into 3 genotypes (A, B, C) with B1-B5 and C1-C5 sub-genotypes [1, 3–7]. Strains of B5 have caused outbreaks in Asian countries since 1997 [8, 9]. EV71 identification has increased since 1998, with continually emerging forms of the sub-genotype C4 in China [10]. Since 2009, outbreaks of EV71 infection in the Republic of Korea have been reported 168 patients with hand, foot, and mouth disease and 92 patients with neurological complications [11].

New vaccine development against human EVs using various technologies [12] includes several EV71 candidates representing live-attenuated virus, inactivated whole virus, recombinant viral proteins, virus-like particles (VLPs), and DNA vaccines that have been evaluated in animals. Furthermore, phase 1 to 3 clinical trials incorporating an inactivated EV71 vaccine have been conducted in Asia [13, 14]; however, no approved effective antiviral drugs or vaccines are currently available, and the inactivated EV71 vaccine was produced by chemical treatment (i.e., formalin inactivation), which could pose a risk of contamination by host cell proteins during downstream purification processes. On the other hand, the alternate platform VLP is noninfectious as it lacks viral nucleic acids yet it preserves EV conformational epitopes.

VLP-based vaccines against a wide range of infectious disease viruses are in various stages of development [15–20], and those against hepatitis B virus and human papillomavirus have been approved commercially for use as human vaccines [21, 22]. Previously, VLPs against poliovirus, an *Enterovirus*, have been generated using EV71 [23], as has co-expression of individual VP0 (36 kDa), VP1 (33kDa), and VP3 (27 kDa) proteins mediated by three recombinant baculoviruses [24]. EV71 VLP has been produced through co-expression of P1 and 3CD, with yield enhancement using exogenous promoters [25, 26].

Relatively low VLP yield represents a major drawback for EV71 VLP vaccine development [25, 26]. Here, to develop a vaccine against the EV71 C4a strain, we generated VLPs through P1 and 3CD expression. We examined whether baculovirus harboring both P1 and 3CD genes driven by the polyhedron or various exogenous promoters, respectively, increased VLP yield. Furthermore, we evaluated the potential of VLP as a vaccine candidate by testing neutralizing antibodies generated by the purified EV71 C4a VLPs.

## Materials and methods

### Cells and media used in VLP production

*Spodoptera frugiperda* (Sf)-9 (American Type Culture Collection, ATCC) and Sf-21 (Invitrogen) were cultured in spinner flasks using SF-900 II medium (Gibco) supplemented with 5% (vol/vol) fetal bovine serum (Gibco) at 27°C. High-Five^TM (^Hi-5) insect cells (Invitrogen) were cultured in spinner flasks using serum free SF-900 II medium (Gibco).

### Generation of recombinant baculoviruses

Entry clones were constructed for P1and 3CD, respectively (S1 Fig). Restriction sites were added to the 5′ (*Bam*HI) and 3′ (*Apa*I, *Sal*I, *Bgl*II, *Nde*I, and *Xho*I) regions of the P1 and (5′, *Bgl*II and *Nde*I; 3′,*Xho*I) 3CD gene fragments, respectively (S1 Fig), which were generated using pEntr-BHRNX (Newgex, Korea) containing attL1 and attL2 as the backbone (S1 Fig). The gene products were digested with *Bam*HI/*Xho*I or *Bgl*II/*Xho*I for P1 or 3CD, respectively.

The gene fragments coding for P1 and 3CD were synthesized from the full-length clones of C4a type (accession No. FJ158600) and B3 type (accession No. AB550334) cloned into pGEM-T easy vectors (Promega), respectively. The gene products were cloned into the corresponding sites in ccdB under the polyhedrin promoter to generate pEntr-P1 or pEntr-3CD as well as the composite pEntr-P1-3CD. The recombinant plasmids were transformed into DH5α *E*. *coli* (Invitrogen). The recombinant baculoviruses Baculo-P1, Baculo-3CD and Baculo-P1-3CD were subsequently generated using the efficient high-throughput recombinant BacHTS system, which incorporates the attR1 and attR2 gateway recombination acceptor sequences for efficient recombination, as described previously [27]. Transfection and selection of the recombinant viruses were performed according to manufacturer instructions of using integrase/exosionase (ElPis, Korea), and cellfectin (Invitrogen), and SF-900 II medium (Gibco), and then the mixture was transfected into Sf-9 cells at 25°C over 4days. Single VLPs were obtained by purification assay.

### Promoter insertion for yield enhancement

The resultant entry clone plasmids were designated pEntr-P1, pEntr-3CD, and pEntr-P1-3CD (S 1). In addition, whereas the polyhedron promoter was retained to drive the P1 gene fragment, various promoter gene fragments were inserted between P1 and 3CD for yield enhancement. The full-length cytomegalovirus immediately early (*CMV-IE*) promoter was PCR-amplified from cytomegalovirus, and *lef3*, *gp41*, and chitinase promoters were PCR-amplified from *Autographa californica* multiple nucleopolyhedrosis virus (Ac*M*NPV), one of the best-characterized baculoviruses containing at least 154 open reading frames [28], respectively. Promoter fragments were inserted into the pEntr-P1-3CD vector, and recombinant Baculo-P1-3CD-*CMV-IE*, Baculo-P1-3CD*-lef3*, Baculo-P1-3CD-*gp41*, and Baculo-P1-3CD-chitinase baculoviruses were generated using the BacHTS system as described above. The graphical diagram depicting the pipeline for the generation of recombinant baculoviruses was indicated in S2 Fig.

### Purification of VLPs by ultracentrifugation

To prove the expression of recombinant baculovirus, VLPs were produced by infecting Sf-9, Sf-21, or Hi-5 cells cultured in SF-900 II medium with the recombinant baculovirus at a multiplicity of infection (MOI) 5. For large-scale production, Hi-5 cells were cultured in 1-L roller bottles (Corning) with 200 mL working volume. The cells were inoculated at 5 × 10^7^cells, grown to 1 × 10^8^, and then infected with EV71 VLP at MOI 5. At 3 days post-infection (dpi), the infected cells were harvested by centrifugation (10,000 rpm for 10 min), and re-suspended in 0.4μL protease inhibitor (Roche) and benzonase (Novagen) in phosphate buffered saline (PBS) to inhibit proteases during infected cell extractions and preserve the integrity of VLP proteins for further sample characterizations. The cell suspension was then disrupted by sonication and centrifuged at 10,000 rpm for 15 min. Subsequently, the supernatant was ultracentrifuged at 25,000 rpm (SW32.1 rotor, Beckman) for 2 h. The pellets were re-suspended in PBS and loaded onto sucrose gradients (10%, 20%, 30%, 40%, and 50% sucrose dissolved in PBS). After ultracentrifugation at 25,000 rpm for 2 h, the milky white band between the interfaces at 35% sucrose was collected. This fraction was used for sodium dodecyl sulphate-polyacrylamide gel electrophoresis (SDS PAGE), western blotting, and transmission electron microscopy (TEM) analyses.

### In vitro assessments

For the *in vitro* cleavage assay, Sf-9 cells were infected at MOI 5 by recombinant Baculo-P1-3CD-*CMV-IE*, Baculo-P1-3CD-*lef3*, Baculo-P1-3CD-*gp41*, and Baculo-P1-3CD-chitinase baculoviruses, respectively, and harvested at 3 dpi. Expression of complete protein was demonstrated by measuring expression of EV71 VLP-gp41 daily until 6 dpi. Hi-5 cells were infected at MOI 5 by recombinant baculovirus. The infected cells were lysed by sonication, and examined by 10% (vol/vol) SDS-PAGE. Western blot analysis was performed using an iBlot gel transfer device (Invitrogen) with Novex gel transfer stacks according to the manufacturer’s instructions. Proteins were detected with mouse anti-EV71 monoclonal antibody 1:10,000 diluted (Mab979, Chemicon International) and mouse anti-EV71 VP1 Mab 1:10,000 diluted (produced in-house) and with goat anti-mouse IgG conjugated with horseradish peroxidase (GenDEPOT) secondary antibody 1:10,000 diluted, following development with GenDEPOT developing reagent.

### Detection and quantification of VLPs

The VLP proteins produced following complete processing and from the total protein in cultured cell lysate were analyzed by Protein 230 Labchip (14–230 kDa) on the Agilent 2100 Bioanalyzer (Agilent Technologies). Samples were prepared by mixing 4μL proteins with 2μL reduced sample buffers containing 4% SDS, florescent dye, and protein markers as internal controls. Samples were incubated in a heating block at 95°C for 10 min, and diluted by adding 84μL distilled water; then, aliquots were loaded onto the microchip. Following electrophoresis, detection was based on laser-induced fluorescence.

### TEM

For TEM analysis, VLPs were suspended in 20μL distilled water and placed on ice. A 300 mesh formvar coated grid was floated on a drop of the VLP suspension for 60s, and excess liquid was removed with a piece of filter paper. The grid was then placed on a drop of 1% uranyl acetate for 10s. Excess stain was removed, and the samples were examined by TEM (Libra-120, Carl Zeiss, Germany) at 120kV.

### Immunization and sera sample collection

EV71 VLP immunogens (purified *gp41*-EV71 VLPs) were prepared by sucrose gradient purification. Groups of 5 female BALB/c mice (5 weeks old) were immunized intramuscularly with 0.1mL 5μg dosages of EV71 VLPs with or without aluminum adjuvant or phosphate-buffered saline (PBS) as a control. The mice were primed, and boosted with the same dosage at week 2 and week 8. Immunized mice were bled every 2 weeks after each immunization, and the serum was collected and was heat inactivated and stored at -70°C and used for immunological analysis.

### Cells, media, and viruses used in VLP immunogenicity assays

The EV71 virus strains used in this study included C4a, B3, B4, and C5 sub-genotypes. EV71 strains were used with different genetic identities: MAL-97-B3 (received from the National Institute of infectious Disease, Japan), Osaka JPN-97-B4 (Accession No. AB059818) and EV71-KOR-C5 (Accession No. HM443663). These virus strains were propagated in Vero (African Green Monkey) cells (ATCC) cultured at 37°C using Dulbecco’s minimum essential medium (GIBCO) containing 5% fetal bovine serum. The virus titers were determined based on the cytopathic effects from the TCID_50_ assay.

### Total specific IgG antibody response

Levels of total anti-EV71 IgG in sera were determined by enzyme-linked immunosorbent assay (ELISA). The EV71 VLP (100ng/100μL) diluted in carbonate buffer (pH 9.6) as coating antigen was used as a coating antigen in 96-well plates at 4°C overnight. After blocking with 5% skim milk in PBS at 37°C for 2 h, the plates were washed with PBS-T(0.5% tween20) three times. Serum samples (diluted 1:200) in dilution buffer (3% skim milk in PBS) was added 100μL into the plates. After 2 h incubation at 37°C, 100 μL horseradish peroxidase (HRP)-conjugated goat anti-mouse IgG (H+L) antibody diluted 1:2000 in dilution buffer was added, and samples were then incubated at 37°C for 1 h. After washed five times, 50 μL of 3,3',5,5'-tetra-methylbenzidine (TMB) solution was added for development. The reaction was stopped by adding 100 μL of 2M H_2_O_4_. Absorbance was measured at 450 nm using a microplate reader.

### Virus neutralization assay

The virus neutralization titer of each serum sample was determined using Vero cells and a standardized TCID_50_ assay according to Liu *et al*. [29]. The EV71 strains used C4a, B3, B4, and C5 sub-genotype, which were obtained from the Korean Centers for Disease Control and Prevention. The analysis of the cross-neutralization titer was used serum sample at 16 weeks. The sera were inactivated at 56°C for 30 min and subsequently diluted from 1:4 to 1:2048 in two-fold serial dilutions (final volume, 50μL), in duplicate, and each dilution was incubated for 1h at 36°C to allow the antibodies to bind to the virus (100 TCID_50_). Next, the Vero cell suspensions (1×10^5^ cells/mL) were added to the virus-serum mixtures in 96-well plates. Virus and cell controls were included for comparison. The plates were incubated at 36°C and examined daily for 5 days for proof of cytopathic effects. Serum with a titer of more than 1:8 was considered positive.

### Statistical analysis

Statistical comparisons among groups were analyzed by one way ANOVA and *t-*test using Graph Pad Prism version 5.00 for windows (Graph Pad software, San Diego California USA, www.grapfhpad.com). A *p*-value less than 0.01 was considered statistically significant.

### Ethical approval

The institutional review board of Korean Centers for Disease Control approved the use of the samples and laboratory animals (approval number: KCDC-024-16-2A).

## Results

### Improvement of yield by promoter

**Fig 1:**
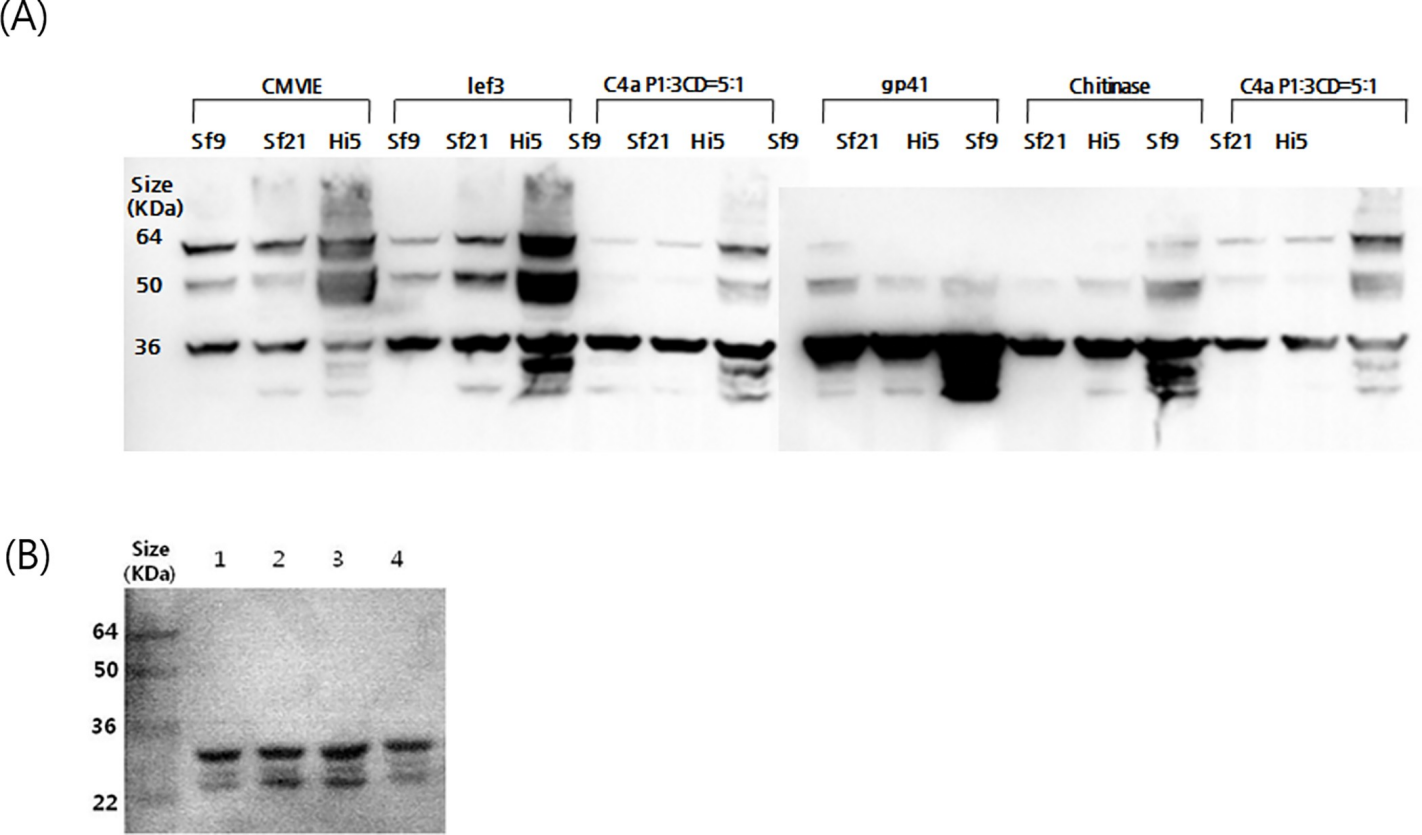
Western blot analysis of cell lysates infected by the Baculo-P1-3CD-gp41 construct. The proteins were separated by SDS-PAGE, electrotransferred to a nitrocellulose membrane, and probed using anti-VP2 MAb (MAb979) as the primary antibody (A). Hi-5 cells used for EV71 VLP large-scale production were infected with Baculo-P1-3CD-gp41 at MOI 5, and protein was analyzed using an anti-VP1 Mab produced in-house (B). Lane1: Baculo-P1-3CD-*CMV-IE* infected cell lysates; Lane2: Baculo-P1-3CD-lef3 infected cell lysates; Lane3: Baculo-P1-3CD-gp41 infected cell lysates; Lane4: Baculo-P1-3CD-chitinase infected cell lysates. The cells were harvested from 3 dpi.

Both EV71 B3 and C4a type after infection with Baculo-P1 with Baculo-3CD at ratios of 5:1, the yield of VP1 production was the highest (S3 Fig). TEM examination EV71 C4a type VLP at ratios of 5:1 showed particles (S4 Fig). However, for the simplicity of production, the formation of VLPs by single infection strategy was further examined. For enhancement of EV71 C4a VLP production as major target, an additional promoter was inserted between P1 and 3CD to express 3CD under the control of the *CMV-IE*, *lef3*, *gp41*, or chitinase promoters. Intracellular VLP yield at 3 dpi after 200mL scale-up culture was quantified using an Agilent 2100 Bioanalyzer (Table 1). Comparison of respective protein yields indicated that maximum yield was obtained from Baculo-P1-3CD-*gp41*, greater than that from Baculo-P1-3CD- *lef3* or chitinase and approximately 4 times higher than that of Baculo-P1-3CD-*CMV-IE* (Fig 1 and Table 1). In Hi-5 cells in 200mL culture, Baculo-P1-3CD-*gp41* produced a VLP yield (< 40 kDa) of 11.3 mg/L (Table 1). Comparison of total viral protein including incomplete processed viral polypeptides was also highest for the *gp41* insertion construct.

**Table 1:** Comparison of VLP yield according to promoter type (units: mg/L).

Predicted MW (kDa)	Promoter type				
	*CMV-IE*	*Lef3*	*gp41*	chitinase	*gp41 extracellular
Viral antigen expected in the EV71 virion (<40kDa)	2.3	10	11.3	7	2.4
Total viral protein (including incomplete processed viral polypeptides)	11.2	41.4	43.1	32.7	5.1

* Concentrated cell supernatant. VLP, virus-like particle; MW, molecular weight

### Optimized production and characterization of VLPs

**Fig 2:**
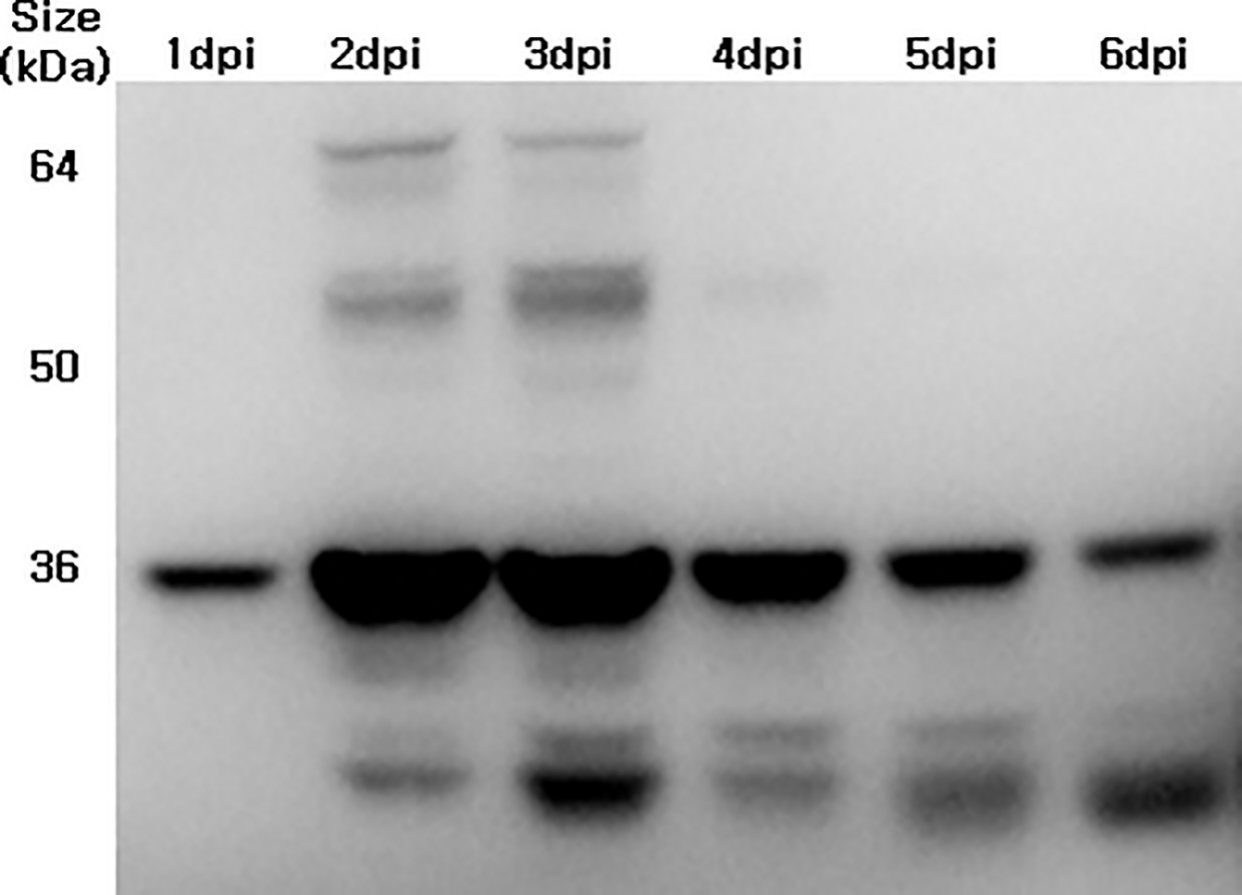
Western blot analysis of Hi-5 cell lysates infected with Baculo-P1-3CD-gp41 at MOI 5 during different times. Hi-5 cell lysate protein was analyzed using an anti-VP2 MAb (MAb979). The cells were harvested from 1–6 dpi.

**Fig 3:**
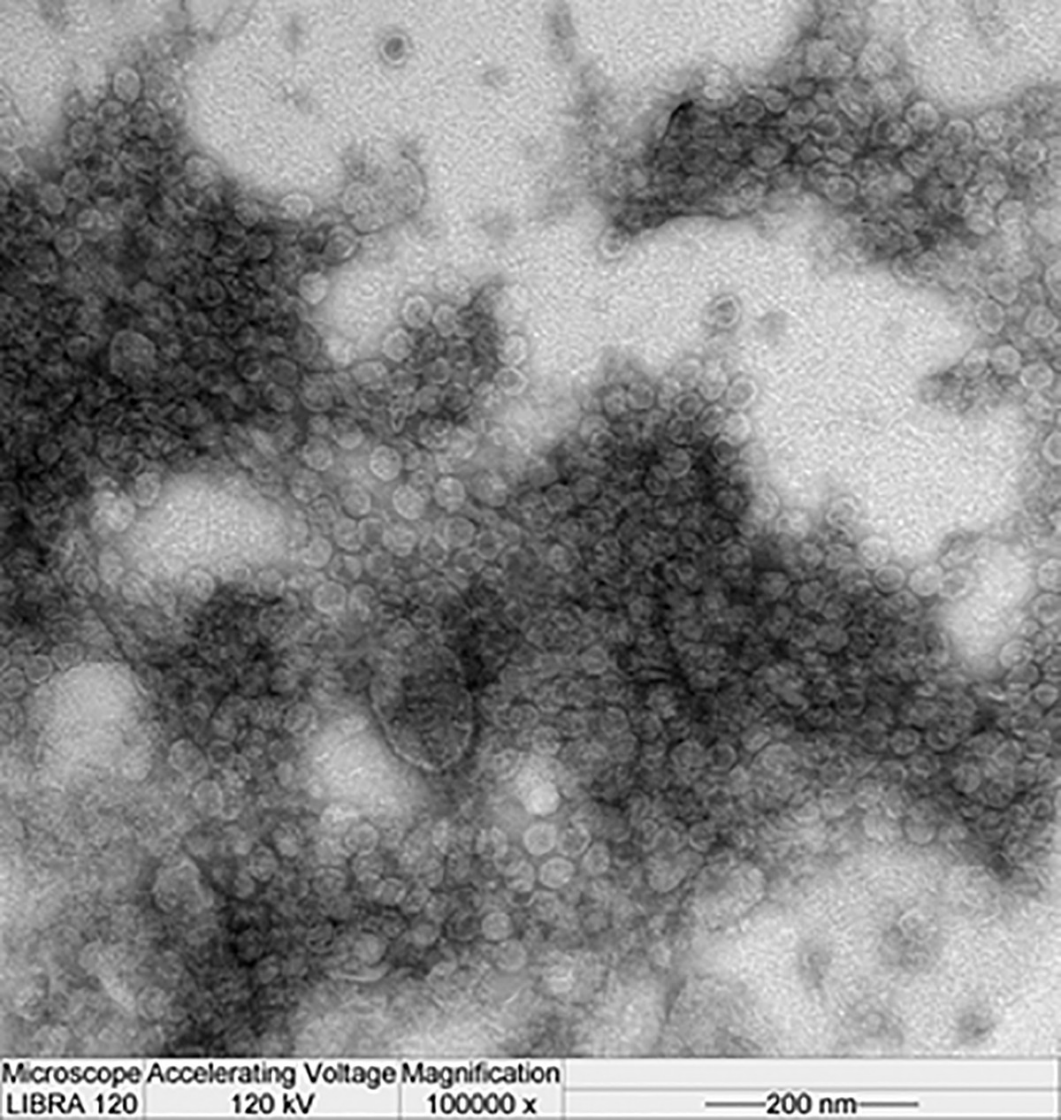
TEM image of EV71 C4a-gp41 VLP produced by Baculo-P1-3CD-gp41. The VLP preparation was purified by sucrose gradient ultracentrifugation.

To examine the P1 expression and cleavage into VP0 (an indicator of VLP assembly) in Sf-9, Sf-21, and Hi-5 cells, these were infected with the 4 promoter variant vectors (MOI 5) at 2.5 × 10^5^ cells/mL (Fig 1). In parallel, Baculo-P1:Baculo-3CD at a 5:1 ratio was infected as the control. Hi-5 infection with Baculo-P1-3CD exhibited improved yield compared with infection of Sf-9 and Sf-21 cells. Expression of Baculo-P1-3CD-*gp41* in Hi-5 cell showed the strongest band intensity indicative of P1 to VP0 cleavage using anti-VP2 Mab (Fig 1A). An anti-VP1 Mab used for examination of VP1 expression (Fig 1B) indicated distinctly higher protein expression of Baculo-P1-3CD-*gp41* in Hi-5 cells. Fig 2 shows that Baculo-P1-3CD-gp41 infection of Hi-5 cells led to correct P1 processing into VP0 and elevated intracellular expression levels at 2 and 3 dpi, along with persistent VLP formation beginning at 2 dpi. Following confirmation of the identity of VP1 (33kDa) and VP0 (36 kDa) bands of EV71 virions on SDS-PAGE and western blot and of the equivalence of the molar ratios of VP0, VP1, and VP3 as predicted [29], we subsequently performed VLP production by infecting Hi-5 cells with Baculo-P1-3CD-*gp41* (MOI 5). The particles were assembled in the infected cells, purified by ultracentrifugation and analyzed. Viral particles observed suggested that the purified sample contained protein whose molecular masses corresponded to those of VP0 (36kDa) (S1 Table). TEM examination (Fig 3) also showed that particles exhibited size and morphology similar to the EV71 intact particle form [29]. These data confirmed that Baculo-P1-3CD-*gp41* infection successfully resulted in the formation of VLP (EV71 C4a-*gp41*) comprising VP1 and VP0 and that these particles were purified by ultracentrifugation.

### Immunogenicity of EV71 C4a-gp41 VLP

**Fig 4:**
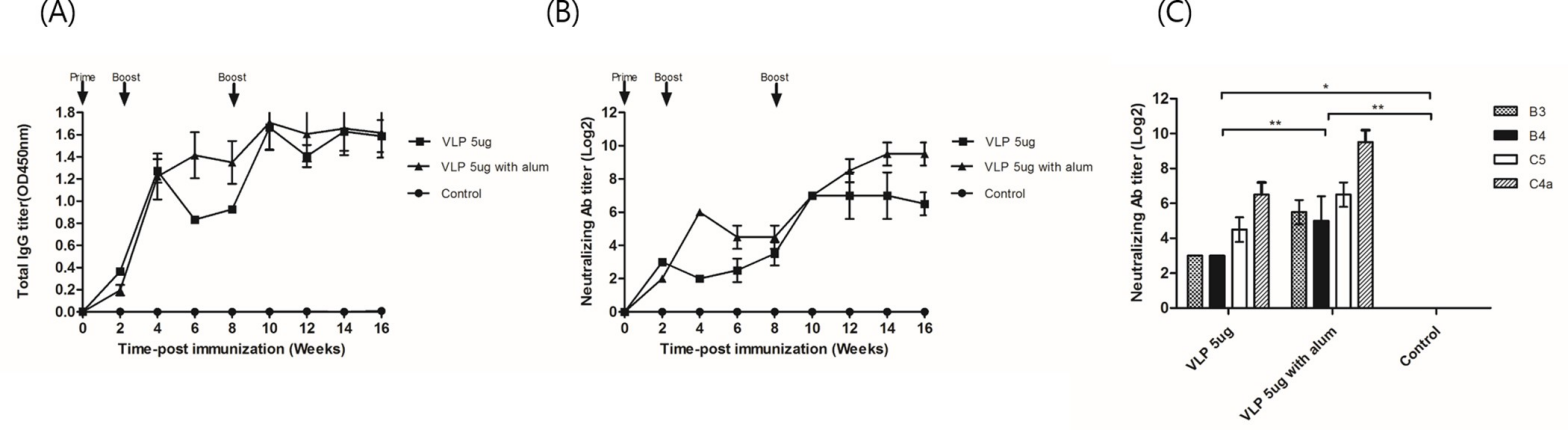
Humoral immune response induced by EV71 C4a VLP-gp41 in mice. The titer of EV71 antibody in each of 5 groups of mice that received EV71 VLP (5μg), VLP (5μg) with alum, or phosphate-buffered saline (PBS) as a control according to immunization schedule as indicated by the arrows. (A) Titers of total IgG antibodies agains EV71 VLP were determined by ELISAs. (B) The titer of the neutralizing antibody against EV71 C4a was assayed by the titer of the serum in a microneutralization assay. The virus titer of EV71 C4a was 100 TCID_50_. (circle, wild type; square, VLP 5μg; triangle, VLP 5μg with alum). (C) Mice received EV71 VLP (5μg), VLP (5μg) with alum, or control and each titer was tested for cross-reactivity in mice against other EV71 subtypes by assaying the titer of the serum in a microneutralization assay. The virus titers of EV71 B3, B4, and C5 were each 100 TCID_50_, respectively (dot bar, EV71 B3 type; black bar, EV71 B4 type; white bar, EV71 C5 type; diagonal line bar, EV71 C4a). These data represent the means of two replicates and error bars indicate SD of the mean. Data statically analysis was performed by one way ANOVA, *p* < 0.01.

We investigated the ability of purified EV71 C4a-*gp41* viral particles to initiate an antigen immune response in mouse, and demonstrated that antisera from all of the immunized groups had total anti-EV71 IgG titer and virus neutralization titers against the EV71 C4a virus (Fig 4A and 4B). The Fig 4A indicated that total IgG reached the plateau at week 4 after the 1^st^ boosting. The EV71 VLP 5μg with adjuvant group induced slightly high levels in comparison to group without adjuvant. The VLP 5μg group added to alum showed a 64-fold increase in the neutralizing titer after first boosting at 4 weeks, and 32-fold increase compare with control after second boosting at 8 weeks (*p*<0.01). In particular, the EV71 C4a-*gp41* VLP 5μg with alum group elicited a neutralizing antibody titer of 1:512 against the EV71 C4a virus that was maintained until the end of the experiment. In contrast, titers in the control group remained at baseline after vaccination. To evaluate cross-reactivity, the sera were subjected to neutralizing assays using other EV71 subtype strains (B3, B4, and C5); results indicated that the antibodies were capable of cross-reacting with EV71 of different genogroups (Fig 4C), with cross-neutralization titer significantly induced in the EV71 C4a-*gp41* VLP 5μg with alum immunized mice in comparison to the control group (*p*<0.01). Also, cross-neutralization titer significantly induced in the EV71 C4a-*gp41* VLP 5μg with alum immunized mice in comparison to the control group (*p*<0.01). The group vaccinated with EV71 C4a-*gp41* VLP increased neutralization titer against EV71 subtype strains C4a and C5 compare with B3, B4 subtype.

## Discussion

In the present study, we generated EV71 C4a VLP encoded P1 and 3CD proteins and performed further analyses to enhance VLP yield. As previous data suggested that VLP assembly required less 3CD than P1 [25], the present study presumed that the VLP yield might be optimized by providing adequate 3CD expression potentially by incorporating different promoters. Therefore, we designed constructs containing P1 driven by the polyhedrin promoter with 3CD under the control of various promoters. We further evaluated whether purified EV71 C4a VLP could be used as a vaccine.

To enhance baculovirus-mediated gene expression in insect cells, we incorporated open reading frames of Ac*M*NPV that serve as stimulators of transcription during early to late gene transcription—*lef3*, *gp41*, and chitinase [28], as well as the *CMV-IE* promoter, known as a strong promoter in mammalian cells and which is also active in insect cells [26], into the EV71 C4a Baculo-P1-3CD expression system. We found that the *CMV-IE* promoter was weaker than the *gp41* promoter, for which a reasonable production yield (approximately 11mg/L) was achieved leading to improved EV71 VLP yield. The high expression level produced by this dual promoter system might be used to expedite the production and purification of VLP vaccines to ensure manufacturing consistency for licensing requirements.

In this study the EV71 C4a-gp41 VLP particles had similar icosahedral structure (by TEM), previously reported [29]. Generally, the morphogenesis of the *Picornaviridae* virus begins with self-assembly of translated P1 polypeptides into a pentamer unit, followed by formation of the empty capsid shell by additional pentamers [30]. Virion formation requires the specific cleavage of the P1 polypeptide by the protease 3CD [25] into the VP0 (VP2+VP4), VP1, and VP3 proteins, which spontaneously assemble into icosahedral procapsids and pack the RNA genome into the provirions [24]. Therefore, in this study we employed three recombinant baculoviruses to express P1, 3CD, or P1 with 3CD simultaneously in order to demonstrate the assembly process. Infection by either Baculo-P1-3CD alone or co-infection at a 5:1 ratio (Baculo-P1:Baculo-3CD) demonstrated the successful expression of both P1 and functional 3CD. Lack expression of 3CD was occurred by inadequate P1 cleavage. Only P1 supply was lead to inadequate VLP assembly that polypeptide size was 95kDa. Notably, co-infection was superior to Baculo-P1-3CD single infection with regard to VP0 production; however, for simplicity of large-scale manufacture and high expression yield we utilized the single Baculo-P1-3CD construct in this study.

EV71 VLP expression differs according to the insect cell type and incubation time. Following optimization, we established a serum-free Hi-5 cell culture system for efficient EV71 VLP production at 3dpi using low MOI seed, which could be easily adapted to a large-scale process. This serum-free medium might avoid the problems associated with bovine serum such as causing allergic reaction in humans and the difficulty of removing the high serum protein content during downstream purification steps.

In this study, we demonstrated the potential of EV71 VLP as a vaccine candidate against EV71 infection by analyzing the neutralizing antibody elicited by purified VLP in mice. These antibodies also exhibited cross-reactivity against the virulent EV71 B3, B4, and C5 sub-genotypes. Thus, immunization with VLP derived from the C4a strain induced potent immune responses as demonstrated by the elicitation of persisting neutralizing ability for other strains. These results suggested that VLP vaccination might provide effective cross-strain protection.

EV71 is the main pathogen associated with HFMD and might lead to disease with severe mortality in children. Thus, to develop an effective vaccine, we generated EV71 C4a VLP using a *gp41* promoter to enhance expression yield. Our results demonstrated that the purified EV71 C4a VLP could be used as candidate for EV71 vaccine development through its ability to generate neutralizing antibodies against EV71 C4a and other strains in mice. Our results could be used to expedite the developmental process for vaccines under clinical trials and to ensure manufacturing consistency for licensing requirements.

## Supporting information

S1 Fig Structure of the pEntr-BHRNX vector and the entry clone constructs used for VLP production. (A) Structure of entry clone constructs; and (B) schematic of the pEntr-BHRNX vector used in VLP production. Recombinant VLPs were produced via the expression of constructs by C4a types. Entry clones for P1, 3CD, or co-expression constructs were prepared for entry into the BHRNX vector containing the polyhedrin promoter. Entry clones were constructed for P1and 3CD, respectively (S1A Fig). Restriction sites were added to the 5′ (*Bam*HI) and 3′ (*Apa*I, *Sal*I, *Bgl*II, *Nde*I, and *Xho*I) regions of the P1 and (5′, *Bgl*II and *Nde*I; 3′,*Xho*I) 3CD gene fragments, respectively (S1B Fig), which were generated using pEntr-BHRNX (Newgex, Korea) containing attL1 and attL2 as the backbone (S1B Fig). The gene products were digested with *Bam*HI/*Xho*I or *Bgl*II/*Xho*I for P1 or 3CD, respectively.
(TIF)

S2 Fig Pipeline for the generation of recombinant baculoviruses. The pipeline for generation of recombinant baculoviruses was indicated graphical diagram as process for the improvement of protein expression by the insertion of different promoters, the validation of VLP expression, purification, and structural and immunological characterization.
QC; quality check.
(TIF)

S3 Fig Western blot analysis of cell lysates infected with Baculo-P1, Baculo-3CD, Baculo-P1 and 3CD, and Baculo-P1 with Baculo-3CD at ratios of 5:1. The Sf-21 cells were infected by the viruses at a total MOI 5 and harvested at 3 dpi. The proteins were separated by SDS-PAGE, electro transferred to a nitrocellulose membrane, and probed using anti-VP2 mAb as the primary antibody. EV71 B3 used as stand for EV71 group B. Lane 1: Baculo-EV71 B3 P1-infected cell lysates; Lane 2: 5:1 ratio mixture of Baculo-EV71 B3 P1 and 3CD-infected cell lysates; Lane 3: Baculo-EV71 B3 P13CD-infected cell lysates; Lane 4: Baculo-EV71 C4a P1-infected cell lysates; Lane 5: 5:1 ratio mixture of Baculo-EV71 C4a P1 and 3CD-infected cell lysates; Lane 6: EV71 C4a P1-3CD-infected cell lysates. The cleavage of the polyprotein P1 into VP0 (36 kDa) by the proteolytic activity of 3CD demonstrated by confirmation of complete viral protein expression. The production levels were compared between Baculo-P1 only, Baculo-P1-3CD only, Baculo-P1 and Baculo-3CD at ratios of 5:1 as indicated by the strongest band intensity.
(TIF)

S4 Fig TEM image of VLPs produced by 5:1 ratio mixtures of Baculo-EV71 B3 P1 and 3CD infected Sf-21 cells. The VLP preparation was purified by sucrose gradient ultracentrifugation. Infection with Baculo-P1 with Baculo-3CD at ratio of 5:1 yielded the highest viral protein production. For confirmation of VLP capsid structure, the infected cells were subjected to lysis by sonication and purified by ultracentrifugation.
(TIF)

S1 Table The characteristics of the EV71 VLPs [29]. (DOCX)
