# Supplementary figures and images for: Efficient expression of enterovirus 71 based on virus-like particles vaccine
Source: PLoS One. 2019 Mar 7;14(3):e0210477. doi: 10.1371/journal.pone.0210477 (PMC6405078; doi:10.1371/journal.pone.0210477)

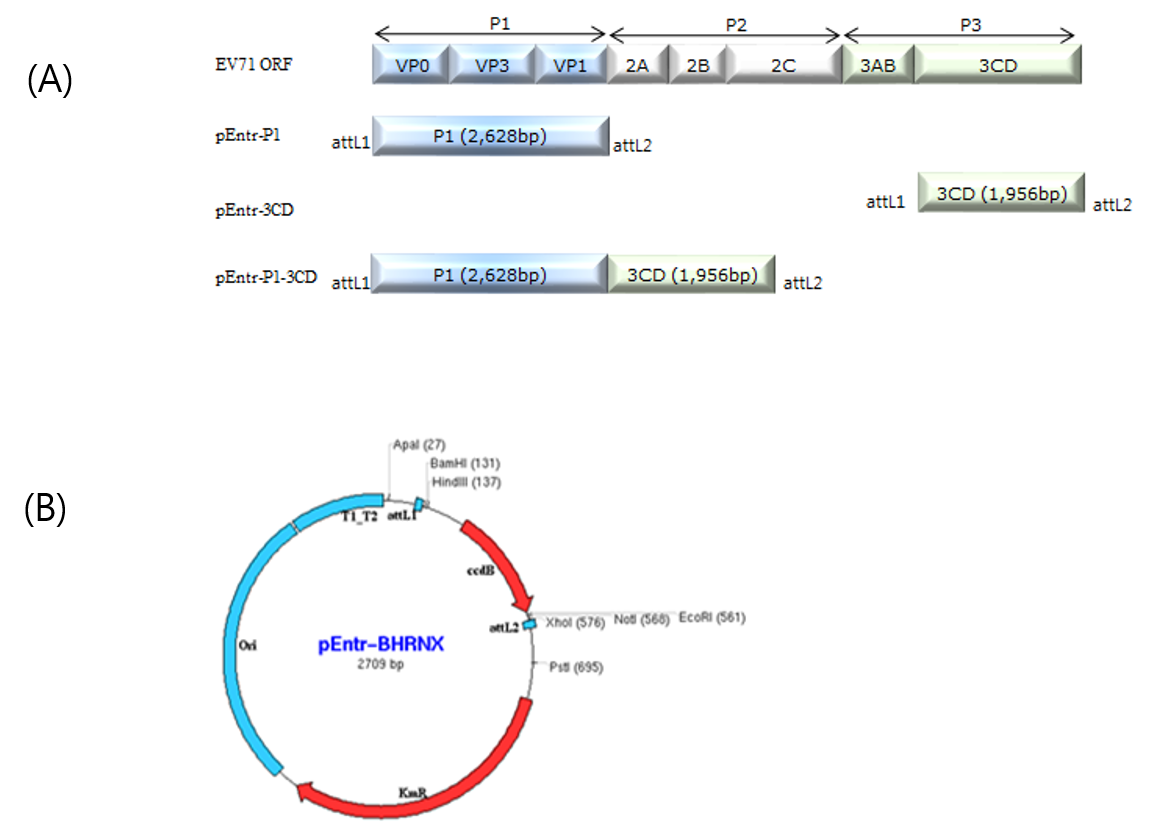

Supplement: S1 Fig — (A) Structure of entry clone constructs; and (B) schematic of the pEntr-BHRNX vector used in VLP production. Recombinant VLPs were produced via the expression of constructs by C4a types. Entry clones for P1, 3CD, or co-expression constructs were prepared for entry into the BHRNX vector containing the polyhedrin promoter. Entry clones were constructed for P1and 3CD, respectively (S1A Fig). Restriction sites were added to the 5′ (BamHI) and 3′ (ApaI, SalI, BglII, NdeI, and XhoI) regions of the P1 and (5′, BglII and NdeI; 3′,XhoI) 3CD gene fragments, respectively (S1B Fig), which were generated using pEntr-BHRNX (Newgex, Korea) containing attL1 and attL2 as the backbone (S1B Fig). The gene products were digested with BamHI/XhoI or BglII/XhoI for P1 or 3CD, respectively. (TIF) [file pone.0210477.s001.tif]

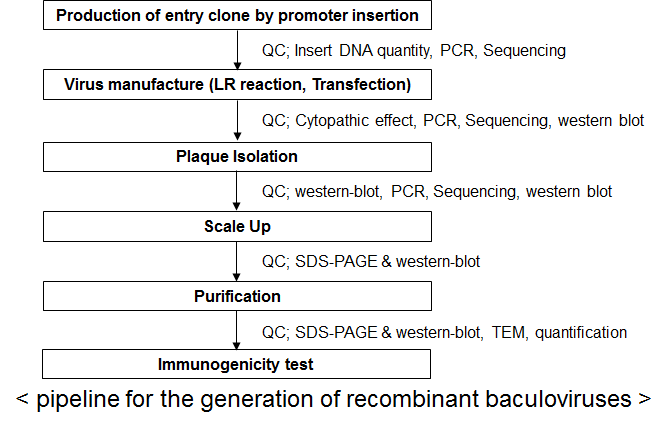

Supplement: S2 Fig — The pipeline for generation of recombinant baculoviruses was indicated graphical diagram as process for the improvement of protein expression by the insertion of different promoters, the validation of VLP expression, purification, and structural and immunological characterization. QC; quality check. (TIF) [file pone.0210477.s002.tif]

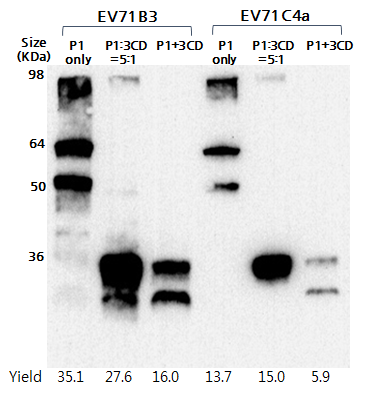

Supplement: S3 Fig — The Sf-21 cells were infected by the viruses at a total MOI 5 and harvested at 3 dpi. The proteins were separated by SDS-PAGE, electro transferred to a nitrocellulose membrane, and probed using anti-VP2 mAb as the primary antibody. EV71 B3 used as stand for EV71 group B. Lane 1: Baculo-EV71 B3 P1-infected cell lysates; Lane 2: 5:1 ratio mixture of Baculo-EV71 B3 P1 and 3CD-infected cell lysates; Lane 3: Baculo-EV71 B3 P13CD-infected cell lysates; Lane 4: Baculo-EV71 C4a P1-infected cell lysates; Lane 5: 5:1 ratio mixture of Baculo-EV71 C4a P1 and 3CD-infected cell lysates; Lane 6: EV71 C4a P1-3CD-infected cell lysates. The cleavage of the polyprotein P1 into VP0 (36 kDa) by the proteolytic activity of 3CD demonstrated by confirmation of complete viral protein expression. The production levels were compared between Baculo-P1 only, Baculo-P1-3CD only, Baculo-P1 and Baculo-3CD at ratios of 5:1 as indicated by the strongest band intensity. (TIF) [file pone.0210477.s003.tif]

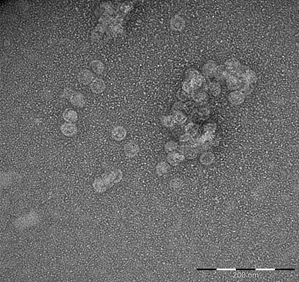

Supplement: S4 Fig — The VLP preparation was purified by sucrose gradient ultracentrifugation. Infection with Baculo-P1 with Baculo-3CD at ratio of 5:1 yielded the highest viral protein production. For confirmation of VLP capsid structure, the infected cells were subjected to lysis by sonication and purified by ultracentrifugation. (TIF) [file pone.0210477.s004.tif]
