# Supplementary material for: Efficient expression of enterovirus 71 based on virus-like particles vaccine
Source: PLoS One. 2019 Mar 7;14(3):e0210477. doi: 10.1371/journal.pone.0210477 (PMC6405078; doi:10.1371/journal.pone.0210477)
Supplement: S1 Table — (DOCX) [file pone.0210477.s005.docx]

**S1 Table. The characteristics of the EV71 VLPs.**

| **Size** | approximately 30-35 nm |
| --- | --- |
| **Morphology** | pentameric icosahedral symmetry |
| **Predicted molecular weight** |  |
| **Viral antigen expected in the EV71 virion** | |
| **VP4** | 8 |
| **VP2** | 28 |
| **VP3** | 27 |
| **VP1** | 33 |
| **Incomplete processed viral polypeptides** | |
| **VP0 (VP4+VP2)** | 36 |
| **VP4+VP2+VP3** | 63 |
| **VP2+VP3** | 54 |
| **VP3+VP1** | 59 |
| **P1 (VP4+VP2+VP3+VP1)** | 95 |
| **Solution compatibility for harvest concentrated virus** | 30% polyethylene glycol or  sucrose gradient or  CsCl gradient |
